# Novel Functions of CD147 in the Mitochondria Exacerbates Melanoma Metastasis

**DOI:** 10.7150/ijbs.52043

**Published:** 2021-01-01

**Authors:** Lixia Lu, Jianglin Zhang, Pingping Gan, Lisha Wu, Xu Zhang, Cong peng, Jianda Zhou, Xiang Chen, Juan Su

**Affiliations:** 1 Department of Dermatology, Xiangya Hospital, Central South University, Changsha, Hunan China.; 2 Hunan Key Laboratory of Skin Cancer and Psoriasis, Xiangya Hospital, Central South University, Changsha, Hunan, China.; 3 Hunan Engineering Research Center of Skin Health and Disease, Changsha, Hunan, China.; 4 Department of Oncology, Xiangya Hospital, Central South University, Changsha, Hunan China.; 5 Department of Plastic surgery, Xiangya Third Hospital, Central South University, Changsha, Hunan China.

**Keywords:** Mitochondria, CD147, Melanoma, invasion, aerobic oxidation

## Abstract

Melanoma is an aggressive form of skin cancer characterized by rapid invasion and metastasis. CD147 is known to be functioning in cell invasion. In this study, we showed that CD147 was translocated from the cell membrane to the mitochondria in advanced melanoma. Melanoma patients with CD147 localized to the mitochondria confer a worse prognosis. The mitochondrial CD147 levels are correlated with the invasion potential of various melanoma cell lines as well as mitochondrial energy metabolism. Depletion of CD147 decreased the activity of mitochondrial complex V. STRING analysis for protein-protein interaction networks (PPIN) in CD147-depleted melanoma cells showed that mitochondrial proteins HSP60 and ATP5B, a subunit of mitochondrial complex V, were node proteins. HSP60 upregulation was correlated with a worse prognosis of melanoma patients. Co-immunoprecipitation (Co-IP) assay indicates that CD147 interacts with HSP60. These data suggested that mitochondrial CD147 may prompt HSP60 to activate ATP5B, thereby promoting the mitochondrial aerobic oxidation and the invasive abilities of melanoma cells. Correlation analysis of the data acquired from patients was helpful to draw a 5-year survival curve for patients who screened positive and negative for mitochondrial CD147. This study unravels the function of CD147 in tumor invasion and highlights it as a potential tumor therapeutic target.

## Introduction

Malignant melanoma (MM) is highly malignant tumor characterized by rapid invasion and metastasis. The incidence rate of melanoma had gradually increased over the past several years. PD1 monoclonal antibody as an immunotherapy, in addition to inhibitors targeting BRAF mutations, is available to many melanoma patients and the overall effective rate needs further improvements. Statistically speaking, the five-year survival rate of patients with advanced MM is only approximately 10%, and the mortality rate accounts for 80% of total mortalities resulting from skin cancer 1, 2. The poor prognosis and high mortality rate render indispensable the exploration of additional therapeutic targets to control the invasion and metastasis of melanoma.

Aberrant energy metabolism is one of the most important signs of a tumor. Warburg pointed out that most tumors exhibit dysfunctions in mitochondrial aerobic oxidation (OXPHOS) and described the increased utilization of glycolysis rather than OXPHOS by tumor cells under physiological oxygen conditions widely known as Warburg effect. Previous studies suggest that MM relies mainly on aerobic glycolysis 3, 4. Enhanced Warburg effect facilitates MM cell growth, invasion and metastasis 4. However, the Warburg effect alone cannot explain subsequent findings among which is the presence of several metabolic signatures in melanoma, all of which are related to cellular oxygen supply. In tumor regions with extreme hypoxia, glycolysis is the dominant metabolic pathway in melanoma; whereas in regions with partial hypoxia, HIF1α expression can inhibit oxygen oxidation. Finally, in tumor regions with sufficient oxygen supply, aerobic glycolysis is the pathway of choice, and mitochondrial oxidation functions well 5. Kallinowski *et al*. determined that mitochondrial oxidation is an essential metabolic pathway in tumor 6, 7. Melanoma has a higher rate of oxygen consumption than other tumors, and this rate is an important marker of aerobic oxidation 8. The mitochondrial oxidative rate is higher in some melanoma cell lines than in melanocytes. Melanoma cells are sensitive to the mitochondrial inhibitors, indicating that mitochondria in melanoma cells still retain their function 7, 9.

CD147 is a transmembrane glycoprotein 10, 11. Experimental investigation indicated that CD147 is an important molecule in the etiology and pathogenesis of numerous diseases. In hypoxic microenvironments, CD147 can promote rapid proliferation of tumor cells through glycolysis. Excessive glycolysis causes elevated extracellular lactic acid, which can damage the host cells 12, 13. A recent study has demonstrated that CD147 binds to NDUFS6, which is localized in the mitochondria and regulates the activity of the mitochondrial complex I 14. In L6 muscle cells, CD147, MCT1, and LDH form a mitochondrial complex that regulates the process of OXPHOS 15. Mitochondrial activity and ROS also modulate CD147 expression 16, indicating that CD147 is directly correlated with OXPHOS obtained. However, no study on whether CD147 affects the invasion and metastasis of MM by regulating aerobic oxidation has been reported. CD147 is known to be redistributed from the plasma membrane to the cytoplasm in advanced MM 14, which is indicative of possible CD147 translocation to the mitochondrial membranes. Thus, we hypothesize that the distribution of CD147 in the mitochondria contributed to malignancy in MM.

This study provided evidence of CD147 translocation from the membrane to the mitochondria in advanced malignant melanoma. Furthermore, MM patients with mitochondrial CD147 suffered worse prognosis of the disease. The localization of CD147 to the mitochondria along with enhanced mitochondrial function promoted invasion in melanoma. Moreover, CD147 interaction with HSP60 prompted ATP5B to influence invasion in melanoma. These results provided new evidence of the role of CD147 in mitochondria and highlight CD147 as a promising therapeutic target to better control melanoma and decrease its mortality rate.

## Materials and methods

### Antibodies

The anti-CD147 [EPR4052] (ab108317), anti-CD147 [MEM-M6/1] (ab666) and anti-Cytochrome C (ab53707) antibodies were purchased from Abcam Biotechnology, Inc. The anti-HSP60 (G0611) and anti-β-actin (E3013) antibodies were purchased from Santa Cruz Biotechnology, Inc., and the anti-GAPDH (6004-1) antibody was purchased from Proteintech, Inc.

### Patients

Eighty-four patients with cutaneous melanoma admitted to Xiangya Hospital between November 2010 and May 2016 were selected for the study. Two dermato-pathologists examined the histological slides independently without being aware of the patient's clinical data. The slides with high pigmentation and technical artifacts were excluded. After the exclusion 47 cases remained and were included in this study. All patients' detail clinicopathological information was tabulated. The clinical variables were age, gender, Breslow thickness, ulceration, Clark stage, histological subtype, mitotic index, tumor sites, lymph node metastasis, distant metastasis, tumor infiltrating lymphocyte, tumor cell infiltration pattern and serum LDH. All patients included in the study provided an informed consent. The use of patients' biopsy slides was approved by the Medical Ethical Committee of Xiangya Hospital and the slides were handled per the Declaration of Helsinki guidelines.

### Immunohistochemistry

Briefly, slides were baked at 65 °C overnight and then dewaxed with Xylene, rehydrated with ethanol gradients and treated with 3% hydrogen peroxide solution for 10 min to inhibit endogenous peroxidase (PO). The slides was immersed in fresh citrate buffer and pressure cooked for 3 min to achieve antigen retrieval. Slides were then incubated with CD147 (1:150, Abcam, USA) and HSP60 (1:300, Santa Cruz, USA) at 4 °C in a wet box overnight. The slides were brought to room temperature (RT) for 30 min on the next day, incubated with universal secondary antibody (anti-mouth/rabbit, 1:200, Zhongshan Company, Beijing, China) for 1 h. Slides were treated with DAB (1:100, Zhongshan Company, Beijing, China) 15 s and then stained with Hematoxylin.

### Immunofluorescence

Cultured cells grown on coverslips were washed with PBS 3 times and fixed with 4% paraformaldehyde for more than 10 min. Fixed cells were incubated with primary antibody at 4°C overnight in a moisturized box after which they were incubated with anti-mouse/rabbit/goat fluorescent secondary antibody for 1 hours at RT. Images were collected using laser confocal microscopy (Thermo Fisher, USA).

### Cell culture, lentiviral and retroviral infections

The human melanoma cell lines SK-MEL-5, SK-MEL-28, A375, G361, MM200 and 293T were maintained in our lab (All cell lines have been identified by professional companies). They were all cultured in DMEM (Bioind, Israel) supplemented with 10% fetal bovine serum (Bioind, Israel). The cell lines were cultured in humidified 37 °C incubators supplemented with 5% CO_2_. Lentiviruses carrying shRNA were generated by transfecting 293T cells with 4 μg of lentiviral vectors encoding shRNA, 3 μg of psPAX2, and 1 μg of pMD2G using Lipofectamine 2000 (Invitrogen). Forty-eight and seventy-two hours after transfection, supernatants containing lentiviruses were collected. Eight hours after infection, the cells were incubated with puromycin (2 μg/ml) for 48 h. The sequence of shRNA was CCGGGCTACACATTGAGAACCTGAACTCGAGTTCAGGTTCTCAATGTGTAGCTTTTT.

### Mitochondrial fraction isolation and Western blot

Mitochondrial proteins were isolated by using the Mitochondria Isolation Kit (Beyotime). Briefly, the cells were collected in centrifugal tube, lysed in mitochondrial dissolution buffer on ice for 10 min and homogenized for 40 times in a glass homogenizer.

Subcellular fractions were obtained by differential centrifugation according to the instructions. The mitochondrial and cytoplasmic components were dissolved in dissolution buffer. The cells were lysed in RIPA buffer on ice for 30 min followed by centrifugation to obtain total proteins. Total protein, mitochondrial and cytoplasmic proteins were detected by WB. Images were obtained by the Bio-Rad imaging system (Bio-Rad, USA).

### Cell viability assay

Digestion was terminated with complete medium, 3×10^3^ cells suspended in 100 μl were seeded in each well of 96-well plates with a volley gun. CCK8 was mixed with the complete substrate at a ratio of 1:10, mixture was added to each well, and the reaction was completed after 2 hours. The plate was read at a wavelength of 550 nm to determine the absorption value. The CCK8 values at 0 h, 24 h, 36 h, 72 h and 96 h were measured according to the above detection methods, and the cell growth curve was generated. Each data point is a representation of 5 replicates.

### Transwell migration assays

Polycarbonate was inserted in transwell chambers (Corning, CA, USA). 5×10^4^ cells suspended in 100 µl in upper chamber and 500 μl 30% FBS in lower chamber. The migration media was DMEM: ECM Gel=6:1 for a total volume of 60 μl. After incubation of 36 hours in cell culture incubator at 37 °C, melanoma cells migrated into the lower chamber and the cells were fixed in 4% paraformaldehyde for more than 10 min and stained with crystal violet for respectively 10-15 min for microscope counting.

### Wound healing assays

A375, G361, SK-MEL-5, MM200 and SK-MEL-28, were seededin6-well culture plate and grown to density up to 90%. Wells were scratched with a sterile 200 μl pipet tip, images were captured at 0 h, 12 h and 24 h using a Nikon microscope to determine the scratch closure.

### Total ATP detection

ATP detection kit (Sangon Biotech, Shanghai, China) was used to examine total ATP in the MM cells. The MM cells were lysed in shaking bed for 10 min and then centrifuged, the ATP level in the cell supernatant was detected by luminometer according to the manufacture's instruction.

### Mitochondrial complex IV and V enzyme activity assay

The activity of complex IV and V were measured similarly. 5×10^6^ cells were lysed and the suspension was homogenized for about 16-20 strokes with a glass pestle in a glass homogenizer. Complex IV and V extraction were subjected to differential centrifugation twice then left to sediment and absorbance was detected by Spectrophotometry. Enzymatic activity was calculated following the formula provided in the manufacturer protocol (See instructions).

### Coimmunoprecipitation (co-IP)

The cells were harvested and incubated in NP40 lysis buffer on ice for 30 min. The lysates were centrifuged at 14,000 rpm for 10 min to remove cell debris. The supernatant was then re-centrifuged at 3000 rpm for 3 min, and mixed with 1.5 μg antibody. After 1 h of incubation, protein A/G agarose beads (Beyotime, Shanghai, China) were added. After rotation overnight at 4 °C, all beads were recovered and rinsed 3 times with NP40 buffer, followed by adding 20 μl of cracking buffer and 50 μl of loading buffer, and then incubated at 95 °C for 10 min.

### Electron microscope

The melanoma cells were fixed with 2% paraformaldehyde and 0.5% glutaraldehyde (A375 and G361) at 4 °C for 12 h and 0.5% osmic acid for 1 h, and then sequentially dehydrated with 30%, 50%, 70%, 80%, 90%, 100% ethanol and 100% acetone for 10 min. The cells were then treated with 100% propylene oxide for 3 times, 10 min/each time, followed by infiltration with the solution of propylene oxide and epon812 for 1 h, and then epon 812 for 12 h. The sample was transferred to the embedding plate and an appropriate amount of epon 812 was added. The embedded samples were incubated at 60 °C for 48 h in an oven and then proceed for ultrathin section. The sections were first treated 1% H_2_O_2_ for 3 times, 10 min/each time, and then blocked with 1% BSA for 15~20 min. Next the sample was incubated with primary antibody at room temperature for 2 h, followed by PBS wash for 3 times, and then blocked with 1% BSA for additional 15~20 min. The sample was then incubated with secondary antibody (1:40 dilution) for 2 h, followed by PBS wash for 10 times, and then rinsed with distilled water for 10 s. After these procedures, the sample was dyed with uranium, and proceeded to TEM imaging.

### Statistical analysis

SPSS statistics 23.0 and GraphpadPrism 7.0 were used for statistical analysis. Kaplan-Meier analysis log-rank method was used for survival analysis including OS and DSS. Cox regression Multivariate analysis model was used to analyze prognosis data. Analysis of variance (ANOVA) was used to compare the differences among various groups and Student *t*-test was employed to compare the difference between two groups. *p*<0.05 was considered statistically significant.

## Results

### CD147 localizes to the mitochondria in advanced MM patients and is associated with a worse prognosis

**Figure 1:**
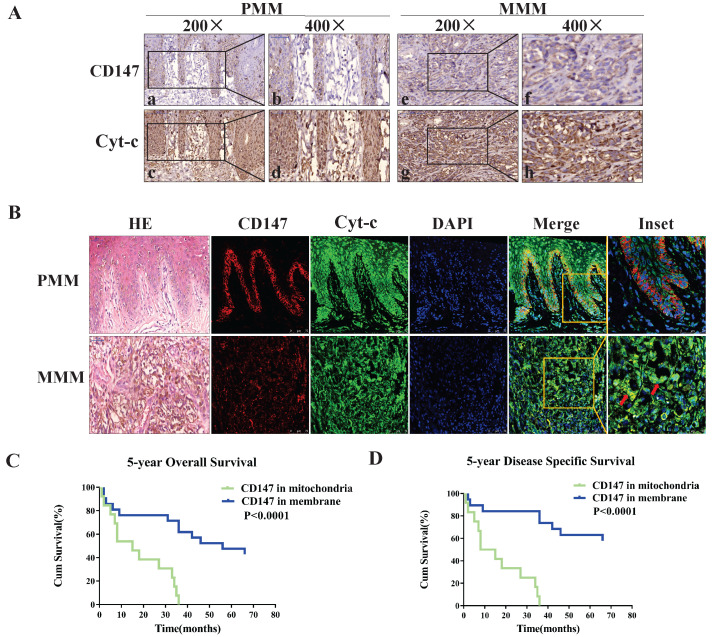
** CD147 localizes to mitochondria in advanced MM patients and correlates with worse disease prognosis.** Expression levels of CD147 and Cyt-c were assessed using IHC and IF. Cyt-c is only expressed in the mitochondria. **(A)** IHC showing CD147 and Cyt-c expression levels in the primary melanoma (PMM, 200× and 400×, a-d) and metastatic melanoma (MMM, 200× and 400×, e-h). (**B**) IF showing CD147 (red) and Cyt-c (green) expression levels in the primary melanoma (row 1) and metastatic melanoma (row 2). (Data from multiple experiments, both *p*<0.0001; ion-rank test). **(C-D)** Kaplan-Meier survival curves showing the correlation between CD147 localization and 5-year OS **(C)** or 5-year DSS (**D**). Cum, cumulative.

Cyt-c is a component of electron transport chain that localizes to the mitochondria, and was used as a mitochondria marker. The expression of CD147 was investigated in 22 primary and 25 metastatic melanoma samples. The immunohistochemical analysis showed that CD147 is localized on the plasma membrane in the primary melanoma (PMM) tissues but in the cytoplasm in the metastatic melanoma (MMM) tissues (Figures 1A). Immunofluorescent staining data showed that CD147 is colocalized with Cyt-c in the mitochondria of MMM (Figure 1B) in addition to the membrane localization in the PMM. The association between CD147 localization and clinicopathological features of melanoma patients was analyzed (Table 1). The localization of CD147 to the mitochondria was positively correlated with Breslow thickness, Clark stage, and tumor-infiltrating monocytes (*p*<0.05), respectively. Multivariate Cox regression analysis was performed to determine whether CD147 mitochondrial localization correlates with survival. The correlations of Clark stage, Breslow thickness, tumor-infiltrating monocytes, and mitotic index with 5-year OS (overall survival) and 5-year DSS (disease specific survival) in these 47 cases were investigated (Table 2). The statistical analysis indicated that CD147 localization, Clark stage, tumor-infiltrating monocytes and mitotic index, but not Breslow thickness (*p*>0.05), were the most significant prognostic markers for 5-year OS and DSS (*p*<0.01, respectively). CD147 localization was also an independent relative factor of prognostic for 5-year OS [relative risk (RR)=4.506, 95% confidence interval (CI)=2.032 to 9.988; *p*=0.000], DSS (RR=0.198, 95% CI=0.086 to 0.458; *p*=0.000). Kaplan-Meier survival curves indicated melanoma patients with CD147 mitochondrial localization conferred a worse prognosis (Figure 1C, 1D).

### CD147 localizes to the mitochondria in MM cell lines

**Figure 2:**
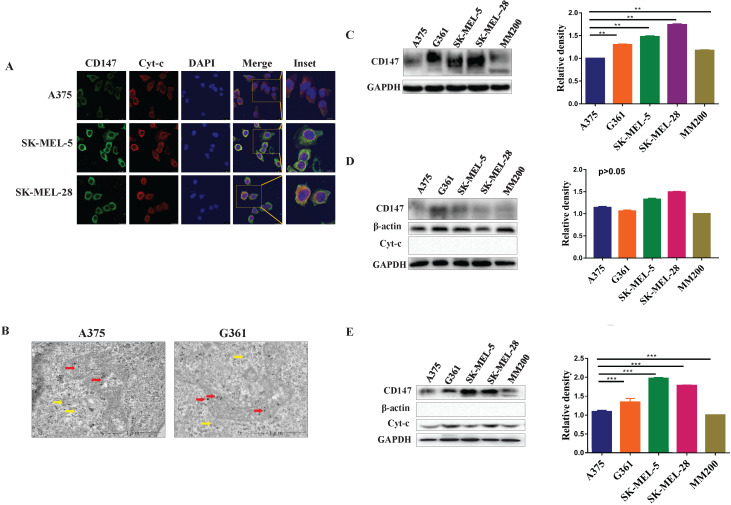
** CD147 localizes to the mitochondria in MM cell lines.** Five MM cell lines were used to further demonstrate the distribution of CD147 in the mitochondria**.** (**A**) The images of HE in PMM and MMM were showed in lane 1. The distribution of CD147 was analyzed in melanoma cells by IF. CD147 (green) (lane 2), Cyt-c (red) (lane 3), DAPI (blue) (lane 4), and merged image (yellow) (lanes 5-6). (**B**) The localization of CD147 in A375 and G361 cell lines were revealed by immunoelectron microscopy. Red arrows, mitochondria; yellow arrows, cytoplasm. CD147 expression was detected by WB in total protein (**C**), cytoplasmic fractions (**D**), and mitochondrial fractions (**E**) in MM cell lines. The level of CD147 in total cell lysates from melanoma cells indicated to be significantly different among the cell lines tested (C, **p < 0.01). The level of CD147 in cytoplasm of melanoma cells was not significantly different among different cell lines (D, *p >* 0.05). The level of CD147 in the mitochondria showed highest in SK-MEL-5, and progressively lowered in SK-MEL-28, G361, A375, and MM200 (E, ***p < 0.001). Data are expressed as the mean ± SD (C-E, n=4).

Immunofluorescence and immunoelectron analysis both showed that CD147 localizes to the mitochondria in different MM cell lines (Figure 2A, 2B). The expression levels of CD147 were further examined by WB in these cell lines. CD147 protein was present in total cell lysates (*p*<0.01, Figure 2C), cytoplasmic fraction (*p*>0.05, Figure 2D), and mitochondrial fraction (Figure 2E) from MM cell lines. CD147 showed highest level in the mitochondria of SK-MEL-5, followed by SK-MEL-28, G361, A375, and MM200 (*p*<0.001).

### The level of mitochondrial CD147 is correlated with the invasion and migration abilities of MM cell lines

**Figure 3:**
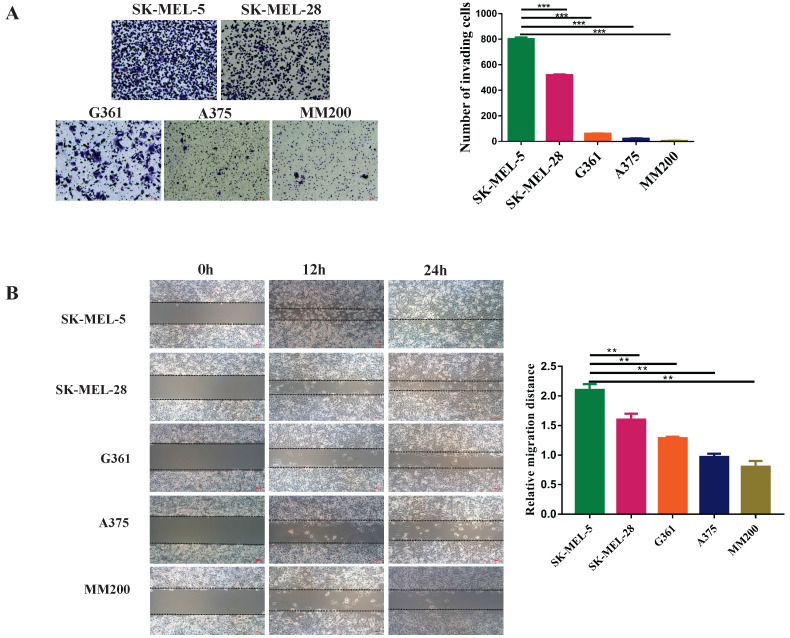
** The invasive and migration abilities of different melanoma cell lines. (A)** The invasive abilities of 5 MM cell lines was detected by transwell assay, including SK-MEL-5, G361, SK-MEL-28, MM200 and A375. The invasive ability of each cell line was evaluated with the number of invading cells. ^***^Indicated *p* < 0.001. **(B)** The wound-healing assay was used to monitor the migration of melanoma cell lines at 0, 12, and 24 h under a microscope. ^**^Indicated *p*< 0.01. Data are expressed as the mean ± SD (A-B, n=3).

The invasion and migration abilities of different MM cell lines were investigated by transwell and wound-healing assays. Transwell assay showed that SK-MEL-5 was the most aggressive melanoma cell line, followed by SK-MEL-28, G361, A375, and MM200 (Figure 3A, *p*<0.001). This was further confirmed by wound-healing assays (Figures 3B). Taken the data shown in Figure 2E together, we concluded that the invasion and migration abilities of cell lines were positively correlated with the CD147 levels in the mitochondria.

### The invasion ability of melanoma cells is associated with the mitochondrial function and may be regulated by mitochondrial complex V

**Figure 4:**
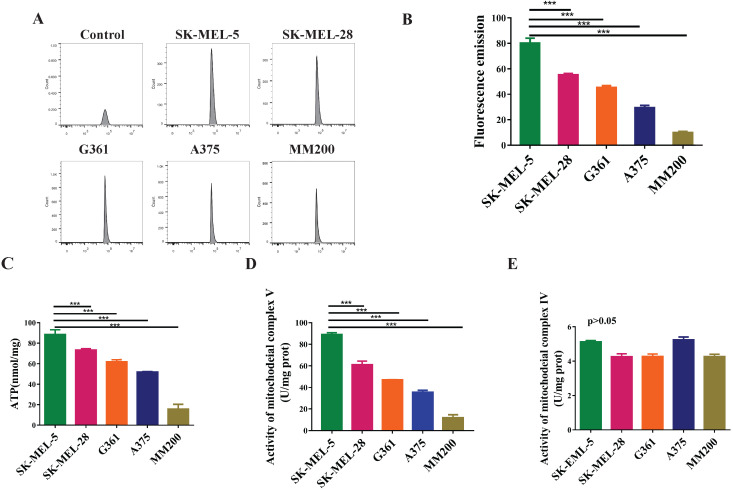
** The mitochondrial functions of different melanoma cell lines.** ROS are metabolites of aerobic oxidation and can be used as an indicator of aerobic metabolism. **(A)** Flow patterns of ROS indicated that the cells were well concentrated and treated. The average values of ROS fluorescence were 80.17, 55.32, 45.2, 29.45 and 9.87 for SK-MEL-5, SK-MEL-28, G361, A375 and MM200 respectively. **(B)** SK-MEL-5 had the highest levels of ROS, of which lower levels were seen in SK-MEL-28, G361, A375, and MM200 (*p* < 0.0001). **(C)** ATP production in different MM cell lines (^***^, *p* <0.001). (**D**) The activity of mitochondrial complex V in the MM cell lines (^***^, *p* <0.001). **(E)** The activity of mitochondrial complex IV showed no significant difference among the MM cell lines tested (*p >*0.05). Data are expressed as the mean ± SD (B-E, n=3).

To evaluate the mitochondrial function in melanoma, we investigated the levels of reactive oxygen species (ROS), total ATP, and the activity of mitochondrial complex IV and V. The data showed highest ROS level in the SK-MEL-5 cells, followed by SK-MEL-28, G361, A375, and MM200 (Figure 4A, 4B, *p*<0.001). A similar trend was observed in total ATP production (*p* < 0.001, Figure 4C) and mitochondrial complex V activity (*p*<0.001, Figure 4D) in these cell lines. However, mitochondrial complex IV activity showed no significant difference among these cell lines (*p>*0.05, Figure 4E). These results indicate a relation between the mitochondrial function and the invasion ability of melanoma cells which may be regulated by mitochondrial complex V but not complex IV.

### CD147 regulates the mitochondrial function through mitochondrial complex V

**Figure 5:**
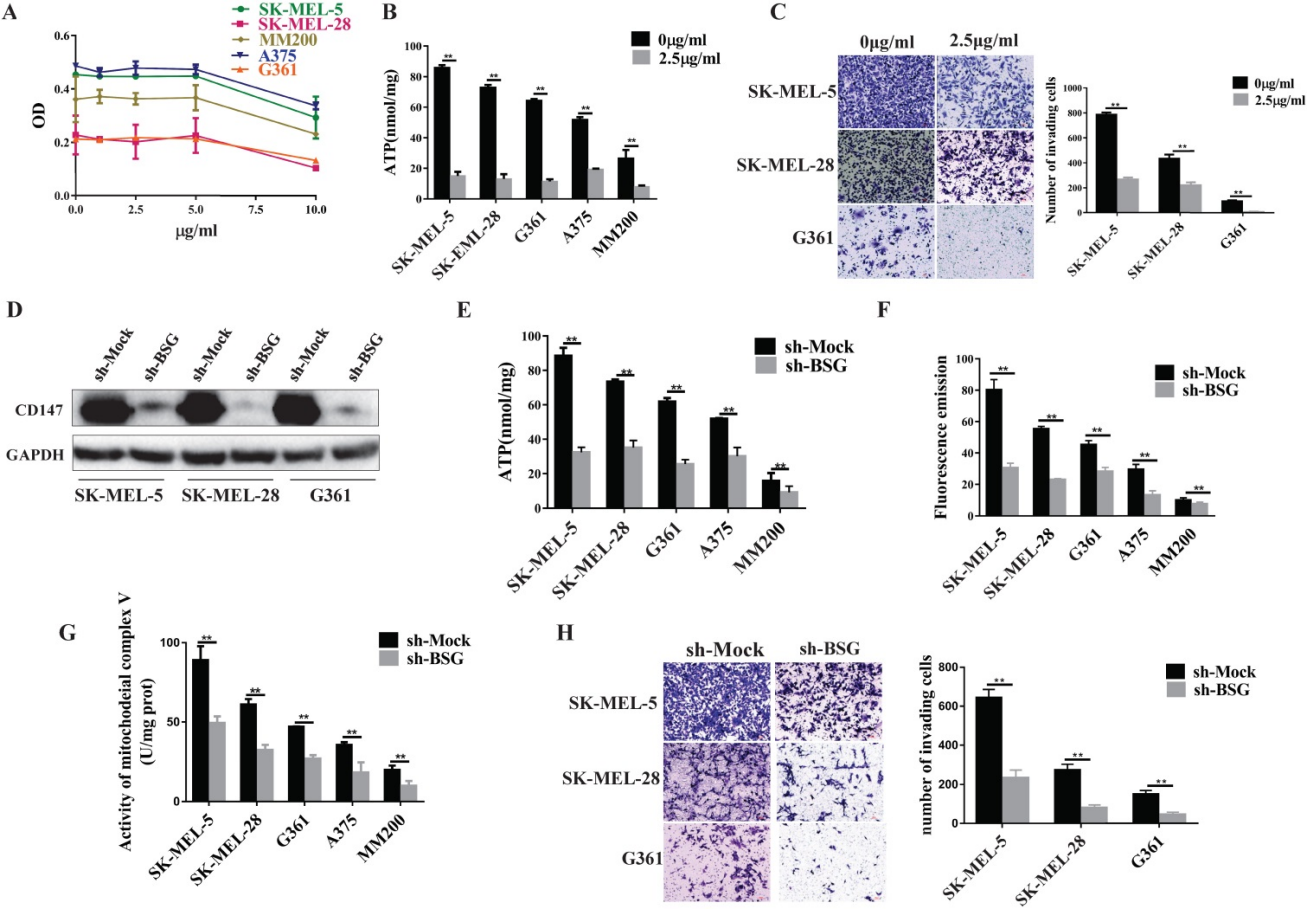
** CD147 regulates the mitochondrial function through mitochondrial complex V**. Oligomycin acts on the mitochondrial complex V and inhibits aerobic oxidation in the mitochondria. (**A**) MTT assay was performed to evaluate the oligomycin effects on the MM cells. (**B**) The ATP production was significantly reduced in all melanoma cell lines treated with oligomycin (2.5 µg/ml) for 24 h compared to untreated control cells (*p* < 0.01). (**C**) Transwell assay showed the invasive ability of the cells treated with oligomycin decreased significantly compared to the untreated control group (*p* < 0.01). Data are expressed as the mean ± SD (B-C, n=3). (**D**) CD147 was knocked down in SK-MEL-5, SK-MEL-28 and G361 cell lines. The protein level of CD147 was assessed by immunoblot (IB) analysis. The ATP production (**E**), ROS levels (**F**), and mitochondrial complex V activity (**G**) were significantly decreased in the CD147 knockdown cells compared to the control cells (*p* < 0.001). (**H**) Transwell assay showed that the invasive ability was significantly reduced in the CD147 knockdown cells compared to the control cells (*p* <0.0001). Data are expressed as mean ± SD (E-H, n=3).

Oligomycin mainly acts on the mitochondrial complex V and inhibits oxidative phosphorylation (OXPHOS). We first optimized the oligomycin concentration in MM cells by MTT assays. The data showed that oligomycin treatment at the concentration of 2.5 µg/ml or 5.0 µg/ml was able to inhibit the cell proliferation effectively. We then chose a lower concentration of oligomycin to inhibit the aerobic oxidation of melanoma (Figure 5A). After treatment with oligomycin at this concentration, the MM cell lines showed decreased ATP levels and invasiveness (*p*<0.01, Figure 5B, 5C). This suggests that the mitochondrial complex V regulates the invasive ability of melanoma.

Next, we went further to investigate whether CD147 regulates mitochondrial function. We knocked down CD147 in the MM cell lines and examined its effects on the mitochondrial function. The data showed that the production of ATP and ROS, and the activity of mitochondrial complex V were significantly reduced in CD147-depleted MM cell lines compared to the control group (Figure 5D-5G). The cell invasive activity also decreased in the CD147 knockdown cells compared to the control cells (Figure 5H).

### CD147 interacts with HSP60 and may regulate the mitochondrial function and melanoma invasion via ATP5B

**Figure 6:**
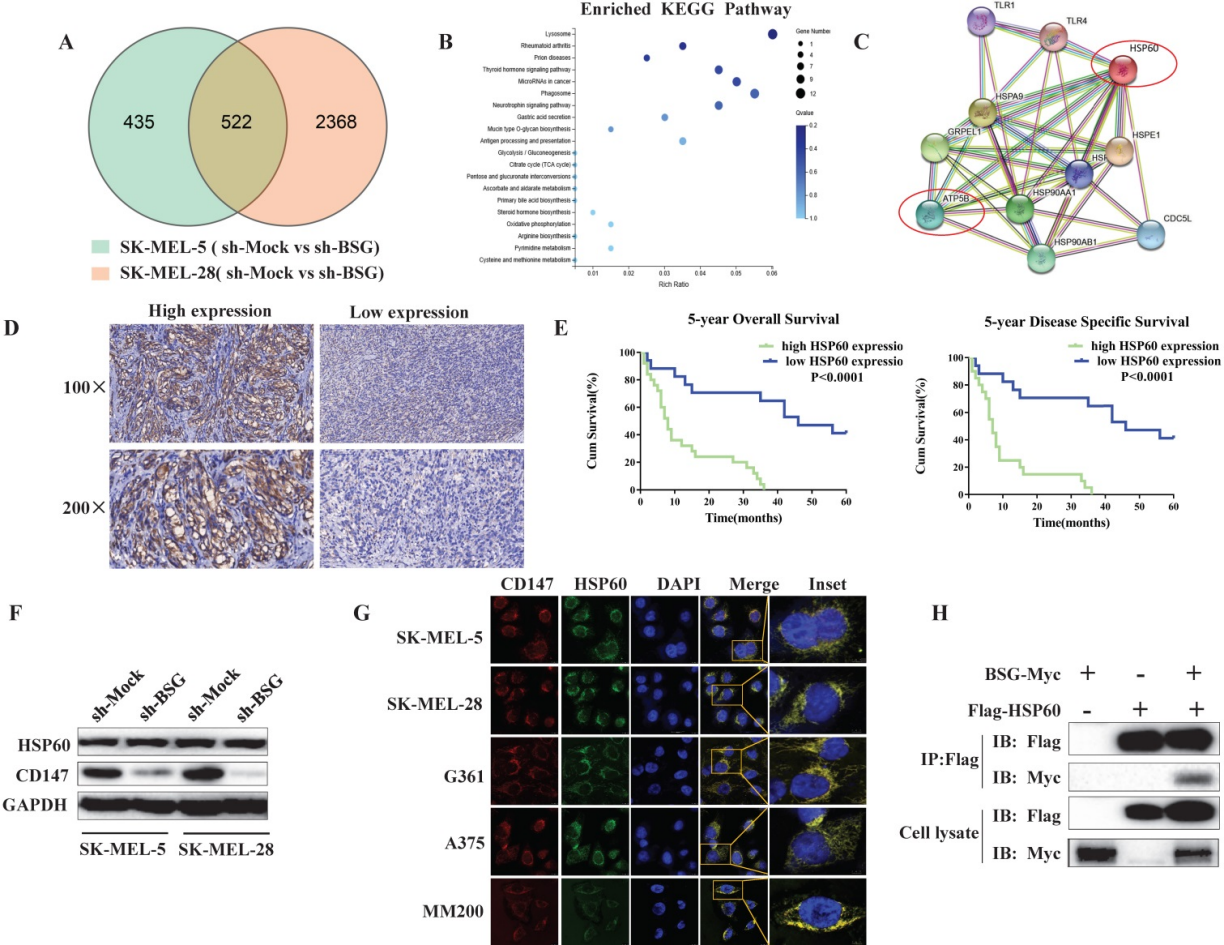
** CD147 interacts with HSP60 and potentially regulates the mitochondrial function and melanoma invasion through ATP5B.** RNA-seq data was used to evaluate the function of CD147 on the gene expression profile in SK-MEL-5 cells. (**A**) The effects of CD147 depletion on gene expression profile. CD147 was stably knocked down in SK-MEL-5 cell lines using two independent shRNA. (**B**) The top 20 positively enriched KEGG pathways are presented in the bubble chart. (**C**) HSP60 and ATP5B are node proteins in the PPIN of CD147-knockdown melanoma cells. The PPIN was established by public website STRING (https://string-db.org/). (**D**) IHC was used to measure the protein expression level of HSP60 in melanoma patients. (**E**) The HSP60 expression is associated with 5-year OS and 5-year DSS. (**F**) The expression of HSP60 is not affected in CD147 knockdown melanoma cells. (**G**) CD147 is co-localized with HSP60 in melanoma cell lines. (**H**) co-IP assays showed that CD147 interacts with HSP60.

To investigate the mechanism by which mitochondrial CD147 facilitates cell invasion in melanoma, we first used RNA-seq to analyze the gene expression profile in the CD147-depleted SK-MEL-5 cells and the control cells (Figure 6A). The top 20 positively enriched Kyoto Encyclopedia of Genes and Genomes (KEGG) pathways are shown in the bubble chart (Figure 6B). We then used STRING to analyze the protein-protein interaction network (PPI) in CD147-depleted melanoma cells. The data showed that HSP60 and ATP5B were node proteins (Figure 6C). ATP5B is a member of mitochondrial complex V. Previous studies confirmed a regulatory role between HSP60 and ATP5B 17, 18, 19, 20.

HSP60 localizes to the mitochondria and plays a key role in mitochondrial function. To investigate whether HSP60 was dysregulated in melanoma, we examined HSP60 expression in melanoma patients by IHC. The data showed that high expression of HSP60 is correlated with a high malignancy of melanoma (Figure 6D). Higher level of HSP60 conferred a worse prognosis for patients, reducing both 5-year overall survival and 5-year disease specific survival (Figure 6E). To investigate whether CD147 regulates mitochondrial function through HSP60, we first examined the expression level of HSP60 in CD147-depleted MM cells. The data indicated that CD147 did not regulate HSP60 expression (Figure 6F). However, CD147 and HSP60 are co-localized in all MM cell lines (Figure 6G). We next performed co-IP by using exogenous proteins. The data showed that CD147 interacts with HSP60 (Figure 6H).

## Discussion

Abnormal energy metabolism in tumors, as well as the effect of such abnormalities on tumor behavior, has long been described 21. Rapid cancer growth leads to a state of anoxia. Under anoxia, cancer cells shut down the OXPHOS of the mitochondria, and switch to glycolysis 22. Glucose is metabolized to pyruvate, which is no longer oxidized by the mitochondrial tricarboxylic acid cycle, but it transformed into lactate, a main intermediate product. To maintain the microenvironment conducive to tumor growth, lactate is transported to the extracellular milieu to generate an acidic microenvironment, which damages the normal cells of the host and further promotes tumor invasion 23, 24. In 1956, Warburg considered that regardless of oxygen content, tumors use mostly anaerobic glycolysis to produce energy 22 and regulate tumor behavior 25. Although the Warburg effect dominates the energy metabolism of melanomas, OXPHOS still plays an important role in melanomas because OXPHOS produces ATP more efficiently than does glycolysis. Although only about 7% of pyruvate enters the TCA cycle in hypoxic cells, OXPHOS provides a large amount of ATP for these cells 25. Scholars have found that besides BRAF mutant cell lines, approximately 35%-50% participant samples can reportedly be described as “High-OXPHOS” 26,27. Therefore, in this study we aimed to determine whether OXPHOS was correlated with melanoma invasion.

Previous studies have reported that the glycolysic rate is higher in melanoma cells than melanocytes, and that the OXPHOS rate of melanoma is lower in melanocytes 28. However, other studies have revealed that melanocytes are more sensitive to glycolysis inhibitors 29, 30, and that he rate of OXPHOS is higher in melanoma cells than in melanocytes 28. Recent research suggests that melanoma have a higher oxygen consumption rate than other neoplasms 7 and is sensitive to mitochondrial inhibitors 31. The above findings indicate that the mitochondria and aerobic oxidation play key roles in melanoma. Our study shows that MM cell lines were sensitive to the mitochondrial complex V inhibitor oligomycin, and the invasive abilities were reduced in the treatment of oligomycin (Figure 5). This finding suggested that mitochondrial function still existed in melanoma and may promote their malignant behavior.

The main function of mitochondria is OXPHOS. The present study indicated that OXPHOS was potentially correlated with melanoma invasion. ATP and ROS are the most important by-products of OXPHOS. Under hypoxic conditions, intracellular ATP is secreted to the extracellular space. ATP is a very efficient extracellular distress signal and a key signaling molecule which is involved in various biological processes 32, 33. Previous studies have reported that the ability of tumor invasion and metastasis decreases after inhibition of ATP synthesis by tumor cells. ATP can also reportedly facilitate tumor invasion. The current work provides evidence that SK-MEL-5 cells had the strongest ability of invasion and also the highest amount of ATP production. After inhibition of ATP production, the invasive ability of SK-MEL-5 significantly decreased.

ROS, another metabolite of OXPHOS, perform a vital role in the processes of tumor progression and metastasis. ROS activate a variety of transcription factors that lead to expression of proteins that promote survival, proliferation, invasion and metastasis. ROS have a great impact on the occurrence and development of tumors 34, 35, 36, 37. The paradox is that ROS also regulate the expression of many tumor- suppressor genes (p53, Rb and PTEN). The relationship between ROS and tumor is hard to explain for numerous reasons 38, 39, 40. High levels of ROS can inhibit tumor growth through activating cell-cycle inhibitors 41, 42. These conflicting claims suggest that the apoptosis of cancer cells is regulated by the same mechanisms that promote their survival. This study revealed that ROS levels were high in the MM cell line with high invasiveness, which indicates that ROS inhibited tumor suppressors to promote invasion in melanoma.

CD147 is a transmembrane glycoprotein that appears to be correlated with tumor invasion and the migration of inflammatory cells. CD147 is highly expressed in many metabolically active diseases, such as melanoma, breast cancers 43, cervical and colon cancers 44, 45. The most important signaling pathway CD147 involved in is regulating MMPs secretion by fibroblasts 46, which degrades the extracellular matrix and provides favorable conditions for tumor invasion 47. For example, CD147 facilitates the growth, invasion of liver cancer by stimulating the production of MMPs 48. In ovarian cancer, the expression levels of vascular endothelial growth factor (VEGF) and MMP9 are downregulated following CD147 knockdown. Meanwhile, CD147 induces the membrane vesicles of NT2/D1 to secrete MMP2 and promote its invasion 49. In Kaposi's sarcoma, CD147 upregulates the VEGF (vascular endothelial growth factor) of endothelial cells to promote invasion 50. This study demonstrated for the first time that CD147 was localized to the mitochondria in advanced MM patients. We also found that ROS and ATP production, as well as the activity of complex V are downstream of CD147 and decreased upon CD147 knockdown. Taken together, these findings suggested that CD147 can facilitate OXPHOS and may promote melanoma invasion through OXPHOS.

HSP60 is a mitochondrial protein that has the most important influence on mitochondrial function among heat-shock proteins 51. HSP60 is a key player in the progress and occurrence of tumors, maintains homeostasis and inhibits apoptosis in tumor cells. Ghosh reported that HSP60 expression influences invasion of advanced prostate cancer. Immunohistochemical analysis shows that the survival is longer in patients with low HSP60 expression compared to those with high HSP60 expression 52. RNA-seq analyses of the effects of CD147 on gene expression in melanoma cell lines, in addition to the STRING analysis of PPIN in CD147-depleted melanoma cells highlighted HSP60 and ATP5B as node proteins. The co-localization and immunoprecipitation results combined with the wound healing and transwell migration findings revealed that mitochondrial CD147 co-localized and interacts with HSP60 to influence melanoma invasion most likely by regulating mitochondrial OXPHOS. These data did not provide evidence that CD147 regulates the levels of HSP60.

In summary, this study was the first to show that CD147 translocalized from the cell membrane into the mitochondria in advanced MM, and that patients with mitochondrial CD147 suffer a worse prognosis. CD147 formed a complex with HSP60 in the mitochondria, which directly regulated the activity of ATP5B in mitochondrial complex V, resulting in the activation of OXPHOS, the increase of ATP production, and finally the invasion of melanoma. This study indicated the important role of mitochondrial CD147 in tumor metastasis and highlights its potential as a therapeutic target in melanoma patients who are positive for mitochondrial CD147.

## Figures and Tables

**Table 1:** Association between CD147 localization and clinic-pathologic characteristics of melanomas

Variables	CD147 expression			*p* value*
	Membrane	Mitochondria	Total	
**Age, years**				0.92
≤59	12 (25.3%)	14 (29.8%)	26	
>59	10 (21.3%)	11 (23.4%)	21	
**Gender**				0.65
Male	12 (25.5%)	12 (25.5%)	24	
Female	9 (21.3%)	13 (27.7%)	23	
**Breslow thickness, mm**				0.0061
≤2.0	14 (29.8%)	6 (12.8%)	20	
>2.0	8 (17.0%)	19 (40.4%)	7	
**Ulceration**				0.80
Present	8 (17.0%)	10 (21.3%)	18	
Absence	14 (29.8%)	15 (31.9%)	29	
**Clark stage**				0.00038
I-II	9 (19.1%)	0 (0%)	9	
III-IV	13 (27.7%)	25 (53.2%)	38	
**Tumor subtype**				0.395
Superficial spreading melanoma	9 (19.1%)	5 (10.6%)	14	
Lentigo malignant melanoma	1 (2.1%)	2 (4.3%)	3	
Acrolentigous melanoma	4 (8.5%)	1 (2.1%)	5	
Nodular melanoma	7 (14.9%)	14 (29.8%)	21	
Unspecified	1 (2.1%)	3 (6.4%)	4	
**Mitotic index**				0.28
≤0.75	18 (38.3%)	17 (36.2%)	35	
>0.75	4 (8.5%)	9 (17.0%)	12	
**Tumor infiltrating monocytes**				0.031
Active	13 (27.7%)	7 (14.9%)	20	
Absence/non-active	9 (19.1%)	18 (38.3%)	27	
**Tumor infiltration pattern**				0.199
Horizontal	12 (25.5%)	3 (6.4%)	25	
Vertical	10 (21.3%)	19 (40.4%)	29	
Both	0 (0%)	3 (6.4%)	3	
**Tumor location**				0.75
Sun-Exposed (head and neck)	2 (4.3%)	3 (6.4%)	5	
Sun-protected (others)	20 (42.6%)	22 (46.8%)	42	
**Lymph node metastasis**				0.75
Present	2 (4.3%)	3 (6.4%)	5	
Absent	20 (42.6%)	22 (46.8%)	42	
**Distant metastasis**				0.75
Present	2 (4.3%)	3 (6.4%)	5	
Absent	20 (42.6%)	22 (46.8%)	42	
**Serum LDH**				0.75
Normal	2 (4.3%)	3 (6.4%)	5	
Elevated	20 (42.6%)	22 (46.8%)	42	

Analysis by χ^2^ test or Fisher's exact test; For the 47 melanoma cases, the median age of the whole group of patients was 57 years (range, 16-80 years).

**Table 2:** Multivariate Cox regression analysis of CD147 localization with 5-year overall survival and disease-specific survival in 47 cases of melanomas

Variables	Overall Survival (OS)			Disease-Specific Survival (DSS)		
	Relative Risk	95%CI	p*	Relative Risk	95% CI	p*
CD147 localization	4.506	2.032-9.988	0.000	0.198	0.086-0.458	0.000
Clark stage	3.062	1.177-7.968	0.022	3.717	1.293-10.686	0.015
Breslow thickness	1.629	0.853-3.111	0.140	1.786	0.908-3.513	0.093
Tumor-infiltrating monocytes	0.299	0.148-0.603	0.001	0.278	0.132-0.582	0.001
Mitotic index	2.366	1.102-5.080	0.027	2.364	1.049-5.329	0.038

Coding of variables: CD147 expression in membrane coded as 1, CD147 expression in mitochondria coded as 2. Breslow thickness was coded as 1, ≤2.0 mm; and 2, >2.0 mm. Mitotic index was coded as 1, ≤0.75; and 2, >0.75; Clark stage was coded as 2, stage Tis, I, II; 3, stage III-V. Tumor infiltrating lymphocyte was coded as 0, absence/non-active and 1, active. Survival was coded as 0, survival; 1, dead and 2, failed to follow-up. *adjustment for age and gender.

## Abbreviations

PMM: primary malignant melanoma
MMM: metastatic malignant melanoma
OS: overall survival
DSS: disease specific survival
BSG: basigin
MMP: matrix metallo-proteinase
ROS: reactive oxygen species
HCC: hepatocellular carcinoma
Cyt-c: cytochrome C
OXPHOS: oxidative phosphorylation
HSP60: heat shock protein 60
KEGG: Kyoto Encyclopedia of Genes and Geomes
PPIN: Protein-Protein Interaction Networks
RR: relative risk
